# 3D-printed microcell for protein NMR at high ionic strengths and small sample volumes

**DOI:** 10.5194/mr-6-157-2025

**Published:** 2025-07-16

**Authors:** Tayeb Kakeshpour, Martin D. Gelenter, Jinfa Ying, Ad Bax

**Affiliations:** 1 Laboratory of Chemical Physics, National Institute of Diabetes and Digestive and Kidney Diseases, National Institutes of Health, Bethesda, MD 20892-0520, USA

## Abstract

Standard solution NMR measurements use 5 mm outer diameter (OD) sample tubes that require ca. 0.5 mL of solvent to minimize “end effects” on magnetic field homogeneity in the active volume of the sample. Shigemi cells reduce the solvent requirement to ca. 0.29 mL. At high ionic strength or at ultrahigh magnetic fields, smaller OD samples are needed to study samples in conductive, radiofrequency-absorbing solvents such as water. We demonstrate an effective and inexpensive alternative for reducing the active sample volume to 0.13 mL by 3D printing ellipsoidal shaped cells that are inserted into 5 mm OD NMR tubes. Static magnetic susceptibility, χ , of printer resin was measured using a simple slice-selection pulse sequence. We found that the χ of water increases linearly with NaCl concentration from -9.05 to -8.65 ppm for 0 to 2 M NaCl. The χ of D_2_O was measured to be -9.01 ppm. The susceptibility difference between the resin ( χ=-9.40 ppm) and water can be minimized by paramagnetic doping of the resin. Such doping was found to be unnecessary for obtaining high-quality protein NMR spectra when using ellipsoidal-shaped cells that are insensitive to susceptibility mismatching. The microcells offer outstanding radiofrequency (RF) and good $B_{o}$ homogeneities. Integrated 600 MHz heteronuclear single quantum coherence (HSQC) signal intensities for the microcell sample in phosphate-buffered saline (PBS) buffer were 6.5±4 % lower than for 0.5 mL of the same protein solution in a regular 5 mm sample tube. The cell is demonstrated for N-acetylated α -synuclein in PBS buffer and for observing tetramerization of melittin at 2 M NaCl.

## 1 Introduction

Nearly all solution NMR measurements are carried out using standard 5 mm outer diameter (OD) sample tubes that require ca. 0.5 mL of solvent to minimize “end effects” on magnetic field homogeneity in the active volume of the sample. The use of a Shigemi cell, which consists of glass with a magnetic susceptibility that is close to that of the selected solvent, can reduce the amount of sample required and thereby limit the cost of expensive protein preparations. However, the tubes are expensive and need to be matched to the magnetic susceptibility of the NMR solvent. The latter can be challenging considering that the susceptibility of water is somewhat temperature-dependent (Schenck, 1996) and increases substantially (becomes less negative) upon the addition of NaCl, whereas it increases by ca. 0.04 ppm in D_2_O versus H_2_O (see below). The sample volume also can be reduced by inserting plugs, of a magnetic susceptibility close to that of the solvent, above and below the active volume of the sample (Barbara, 2009).

Magnetic susceptibility measurements of ionic solutions are often carried out using a magnetic field that oscillates at frequencies ranging from ∼50 Hz to low megahertz (Tsukada et al., 2006; Gutiérrez-Mejía and Ruiz-Suárez, 2012). However, for magnetic resonance purposes, it is the magnetic susceptibility measured in a static magnetic field that is relevant to distortions in field homogeneity (Sangal et al., 2023) and to our efforts to develop a small-volume sample cell for protein NMR.

We describe a simple method for measuring magnetic susceptibility of solid material in a high-resolution NMR spectrometer and the development of a 3D-printed microcell that can be inserted into a regular 5 mm NMR tube. The resin that we used for 3D printing at high resolution (25 µm ) has a reported magnetic susceptibility of χ=-9.34 ppm (Sangal et al., 2023), which is well below that of water. However, by printing the cell with a spherical (Hizawa et al., 2017) or ellipsoidal geometry, magnetic field homogeneity within the cell becomes insensitive to the susceptibility mismatch between the solvent and the printer resin (Schenck, 1996; VanderHart, 1996). The shape of our cell is perturbed by a narrow-diameter, cylindrical access port that is needed to fill it with the NMR sample. Magnetic susceptibility mismatching effects, resulting from the deviation of a perfect ellipsoidal shape caused by this access port and by the finite resolution of the printer, can be minimized by paramagnetic doping of the printer resin with organic paramagnetic salt (Evans, 1959) but is found unnecessary for routine applications. The cells are reusable, but the cost of printing such cells is minimal and recycling them therefore may not be necessary.

We note that susceptibility mismatching for non-ellipsoidal shapes can also be obtained by introducing nearby suitably shaped small “compensation structures” of different magnetic susceptibility, that effectively shim the sample to homogeneity for microfluidic applications (Ryan et al., 2014). Alternatively, the magnetic susceptibility of an aqueous solution can be increased by the addition of Eu^3+^-complexed diethyl-triamine pentaacetate (DTPA), which due to the short Eu^3+^ electron T_1_, has minimal broadening effects on other solutes (Hale et al., 2018) but is restricted to cases where the solvent susceptibility is more negative than the surrounding material, which does not apply for 3D-printer resin.

The microcell introduced by us for biological NMR spectroscopy purposes is particularly useful when the available sample quantity is limited or when high ionic strength is required. High ionic strength lowers the quality factor ( Q ) of radiofrequency (RF) coils and thereby negatively impacts NMR sensitivity, an effect that scales steeply with frequency (Ugurbil, 2018). RF penetration of water has been studied extensively for applications to magnetic resonance imaging, where it impedes the observation of tissue far from the body surface (Roschmann, 1987). RF absorption at high and ultrahigh magnetic fields also challenges solution ^1^H NMR spectroscopy, where the use of pulses that are compensated for both offset and RF inhomogeneity (Freeman et al., 1980; Xia et al., 2017) becomes essential, in particular for the vast majority of advanced experiments that include multiple 180° pulses (Manu et al., 2023). The use of the 3D-printed microcell greatly reduces problems with probe detuning, lowering of Q , and RF absorption. Consequently, use of the microcell results in short ^1^H pulse widths with superior RF homogeneity, even at elevated ionic strength.

Measurements of magnetic susceptibilities of materials with application to magnetic resonance imaging (MRI) as well as microfluidic NMR technology have commonly employed MRI scanner equipment, and elegant methods applied to a wide range of materials were presented by Wapler et al. (2014). The same approach was recently used to measure magnetic susceptibility of 3D-printed materials (Sangal et al., 2023); we demonstrate simple methods for deriving magnetic susceptibility differences between ionic solutions and printer material in a high-field solution NMR magnet. We also demonstrate that while using only 130 µL of solvent, the sensitivity in common multi-dimensional NMR experiments, such as the gradient-enhanced heteronuclear single quantum coherence (HSQC) (Kay et al., 1992), is comparable to what is obtained on a regular 500 µL sample. In another application, we show that high-quality spectra can be obtained for 90 µg of recombinantly expressed and chemically amidated melittin at 2 M NaCl concentration. Milligram-scale expression and purification of this uniformly ^15^N-enriched peptide in its post-translationally modified state, which tetramerizes in a salt-dependent manner, is expensive and very labor-intensive (Gelenter and Bax, 2023).

**Figure 1:**
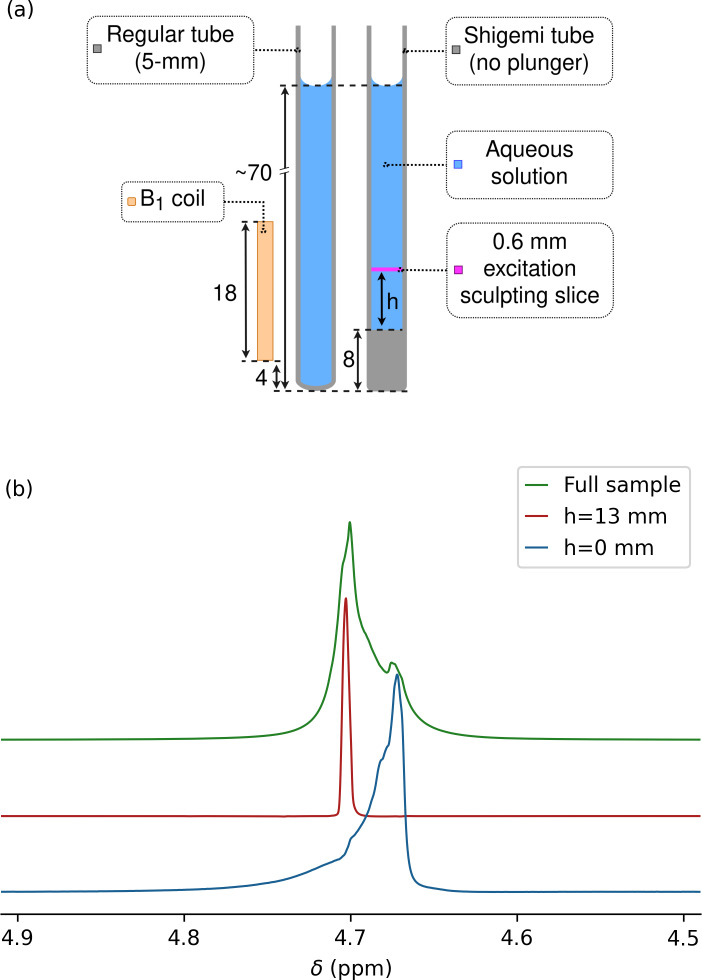
Measurement of magnetic susceptibility in a Shigemi sample tube. **(a)** Prior to measurements on this sample, the magnetic field homogeneity was optimized for a regular 5 mm NMR sample tube filled to the same height of 70 mm above the bottom of the tube and inserted to the same depth into the probe head. Dimensions are in millimeters. A thin slice (pink) through the sample at height h above its bottom was selected by excitation sculpting. **(b)** HDO resonances for the Shigemi sample tube containing 0.3 % H_2_O / 99.7 % D_2_O (no plunger). The glass–liquid interface of the Shigemi cell was positioned 5 mm below the center of the receiver coil using the shim settings of a regular 5 mm sample tube filled to the same total height with the same solution and inserted to the same depth into the NMR probe (see left half of panel **a**). Overlaid are spectra recorded for the entire sample (green) and for 0.6 mm thickness horizontal slices through the Shigemi cell centered at h=0 mm (blue) and at h=13 mm (red) using excitation sculpting while applying z gradients. Spectra were scaled to show the same intensity.

## 2 Results and discussion

### 2.1 Measurement of magnetic susceptibility

For all materials pertinent to high-resolution solution NMR, the induced magnetization M depends linearly on the applied magnetic field $H_{o}$ :

1 $$M=\chi H_{o},$$

where χ is the volume magnetic susceptibility, often expressed in units of parts per million (ppm); i.e., χ is negative for diamagnetic media and positive for paramagnetic substances. The magnetic flux density, $B_{o}$ , is directly proportional to $H_{o}$ :

2 $$B_{o}=\mu H_{o},$$

where the magnetic permeability μ corresponds to $\mu =(1+\chi )\mu_{0}$ and $\mu_{0}=4\pi 10^{-7}H/m$ is the permeability of vacuum. In an NMR sample, the magnetic flux density corresponds to the sum of the applied magnetic field $H_{o}$ and the integral of the magnetic field contributions from the induced magnetization over all sample volume elements at locations r relative to the point of interest (Barbara, 1994). For a cylindrical sample of infinite length, with its axis parallel to a homogeneous applied magnetic field, the integral over all space within the cylinder is uniform across all locations, resulting in

3 $$B_{o}=\mu_{0}(1+\chi )H_{o}.$$

Magnetic flux is conserved at any interface orthogonal to $H_{o}$ between two media with magnetic susceptibilities $\chi_{1}$ and $\chi_{2}$ . For the example of a Shigemi sample cell, where the bottom segment of the cylindrical tube consists of glass with susceptibility $\chi_{g}$ and $\chi_{s}$ is the solvent susceptibility above it (Fig. 1a), the flux density at the interface is given by

4 $$B_{o}=\mu_{0}[1+(\chi_{g}+\chi_{s})/2]H_{o}.$$

Away from the interface, the integrated contributions to $B_{o}$ from volume elements below and above the interface depend on the height above the interface as well as the transverse location, with values converging to $B_{o}=\mu_{0}(1+\chi_{s})H_{o}$ at distances above the interface that are large relative to the tube inner diameter, D . Therefore, when selectively observing the solvent signal from a very thin slice perpendicular to the sample axis at height h above the interface (Fig. 1a), a narrow signal at a frequency $\delta (h)=[1+(\chi_{g}+\chi_{s})/2]\delta_{o}$ is observed for h≪D , with δ(h) approaching $(1+\chi_{s})\delta_{o}$ for h≫D and a complex line shape for slices taken at intermediate values (Barbara, 1994).

Hence, for a solution above the solid glass of a Shigemi cell with a total solvent height that is large relative to both D and the height of the receiver coil, the line shape corresponds to δ(h) contributions ranging from $[1+(\chi_{g}+\chi_{s})/2]\delta_{o}$ to $(1+\chi_{s})\delta_{o}$ . In other words, the total width of the line shape at its base corresponds to half the difference between $\chi_{g}$ and $\chi_{s}$ in units of parts per million. The above analysis does not take into account the $B_{0}$ gradient inside the NMR tube that is caused by the large magnetic susceptibility mismatch at the bottom of the tube. However, when assuming that the magnetic susceptibility of the Shigemi glass is much closer to that of water than air, shimming on a water-filled sample tube inserted to the same depth into the probe can be used to remove this $B_{0}$ gradient to the first order.

### 2.2 Measurement of magnetic susceptibility in a high-resolution NMR magnet

Careful measurements of solvent susceptibility in a high-resolution magnet that used the gas phase of tetramethylsilane (TMS) as an internal reference have been reported by Hoffman (2020, 2022). Here, we describe a different approach that also permits susceptibility measurements of solid objects. First, we demonstrate the method for measurement of the susceptibility of the glass bottom section of a Shigemi cell.

Bruker's topshim program was used to minimize magnetic field inhomogeneity of a regular 5 mm sample that contained a 1 mL solution of a variable NaCl concentration in 97 % $D_{2}O$ / 3 % H_2_O that was inserted into the probe to have its bottom ∼13 mm below the center of the receiver coil. Then, without changing the shim settings, the _1_H line shape on the same solvent composition was observed for a Shigemi sample tube inserted to the same depth into the probe head. Because the Shigemi tube had an 8 mm bottom segment of solid glass, the flat solvent–glass interface is then located 5 mm below the center of the receiver coil (Fig. 1a). If $\chi_{g}$ were identical to $\chi_{s}$ , the same perfect line shape would be expected, but with approximately 15 % lower intensity because the bottom ∼15 % of the receiver coil was now filled with solid glass from the Shigemi tube.

For the 97 % D_2_O / 3 % H_2_O Shigemi tube sample, a pronounced upfield shoulder was observed (Fig. 1b, green), indicative of susceptibility mismatching. Excitation sculpting (Stott et al., 1995) (Sect. A1) while applying a pulsed z gradient was then used to select a slice of 0.6 mm thickness, centered at the glass–solvent interface, i.e., selecting a ∼0.3 mm solvent layer just above the interface. A resonance for this layer was observed that was 0.032 ppm upfield from the most intense segment of the solvent obtained with a 30° pulse without slice selection (Fig. 1b). When selecting a slice at a height of 13 mm above the interface, the signal (Fig. 1b, red) coincided with the maximum of the resonance obtained without slice selection. Therefore, the difference in frequency between the red and blue resonances provides a good measure for the difference between $(\chi_{g}+\chi_{s})/2$ and $\chi_{s}$ . The precision at which the frequency at the glass–water interface can be measured is impacted by the slice thickness of the selected solvent layer, which is subject to lateral gradients that increase with slice layer thickness and towards the edges of the slice. By varying the position of the center of the slice from ∼0.2 mm below the interface to ∼0.2 mm above the interface, increased total intensity with a strong downfield shoulder is observed. The upfield edge of this line shape (Fig. 1b, blue) remains invariant to the precise position of the selected slice and represents the true $(\chi_{g}+\chi_{s})/2$ value, which can be determined at a precision of ∼0.02 ppm.

Repeating the same measurement but using 97 % H_2_O / 3 % D_2_O and strongly mistuning the probe head to reduce radiation damping showed a shoulder that was 0.0183 ppm closer to the frequency observed 8 mm above the center of the coil (i.e., h=13 mm; Sect. A2). Accounting for the solutions not being fully deuterated or protonated, their difference in static magnetic susceptibility then equals $\chi_{H_{2}O}$ - $\chi_{D_{2}O}=2\times 0.0183\times (100/94)=0.04$ ppm. The widely used literature value for $\chi_{H_{2}O}$ is -9.05 ppm (Sangal et al., 2023), yielding $\chi_{D_{2}O}=-9.01$ ppm, which is in fair agreement with Hoffman's measurements (Hoffman, 2022). Using $\chi_{D_{2}O}=-9.01$ ppm as a reference, the susceptibility of the Shigemi glass used in our measurements is $\chi_{g}=-9.08$ ppm.

**Figure 2:**
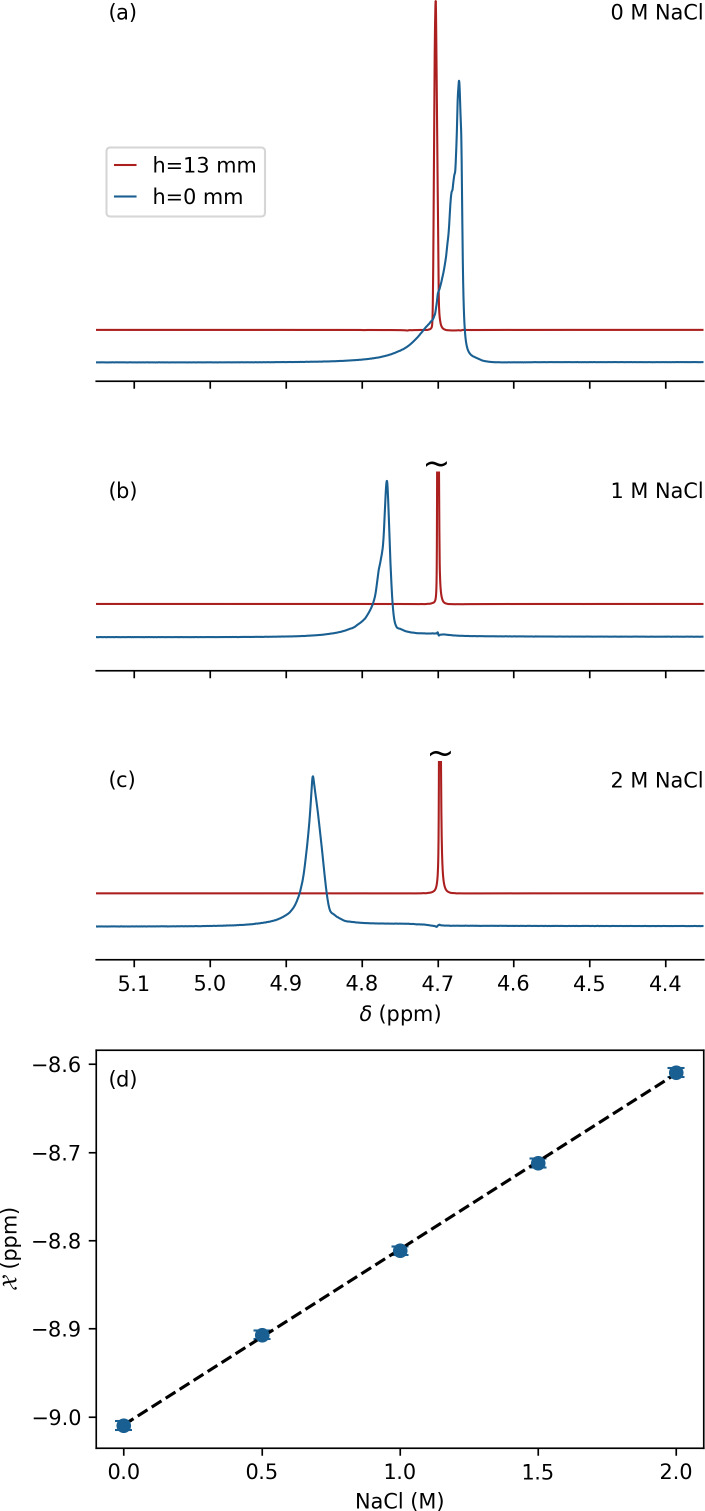
Effect of salt on magnetic susceptibility of 97 % D_2_O. Resonances shown correspond to a ca. 0.6 mm thick slice through a Shigemi tube centered at h=0 (blue) and h=13 mm (red) above the glass bottom of the Shigemi sample cell using the same protocol as for Fig. 1b at **(a)** 0 mM NaCl, **(b)** 1.0 M NaCl, and **(c)** 2.0 M NaCl. **(d)** Magnetic susceptibility of D_2_O as a function of NaCl concentration using $\chi_{D_{2}O}=-9.01$ ppm as a reference. The intensities of the slices at h=0 are upscaled ca. 16-fold to account for the 50 % smaller aqueous volume and the strong magnetic field inhomogeneity in these lower slices.

### 2.3 Salt dependence of water magnetic susceptibility

The effect of dissolved NaCl on magnetic susceptibility of water is important to protein NMR. We therefore repeated the above measurements of the 97 % D_2_O / 3 % H_2_O Shigemi tube sample after the addition of 0.5, 1, 1.5, and 2 M analytical-grade NaCl (Sigma-Aldrich) to the solvent (Fig. 2a–c), showing a linear increase in solvent susceptibility with salt concentrations over the range of 0 to 2 M: $\chi_{D_{2}O+\mathrm{NaCl}}=([\mathrm{NaCl}]\times 0.2$ –9.01) ppm, where [NaCl] denotes the concentration molarity in units of moles per liter (Fig. 2d).

### 2.4 Susceptibility and paramagnetic doping of 3D-printer resin

Clear V4 resin was used because it enabled the highest precision (25 µm resolution) of printing on a Formlabs Form3+ 3D printer available in our laboratory, and its optical transparency facilitated sample handling for 3D-printed microcells. The susceptibility of the printed Clear V4 resin was measured in the same manner as described above for the Shigemi tube. A cylindrical plug of 2 cm length was printed and pushed to the bottom of a standard 5 mm NMR sample tube that was prefilled with 0.7 mL 1 % H_2_O / 99 % D_2_O, such that the top of the plug was again 5 mm below the center of the receiver coil once inserted into the magnet. Shimming of the magnetic field was carried out on a sample without the plug filled to the same level using ∼1 mL of the same solvent and inserted into the probe head at the same depth, i.e., with the bottom of the sample tube at 25 mm below the center of the RF coil.

**Figure 3:**
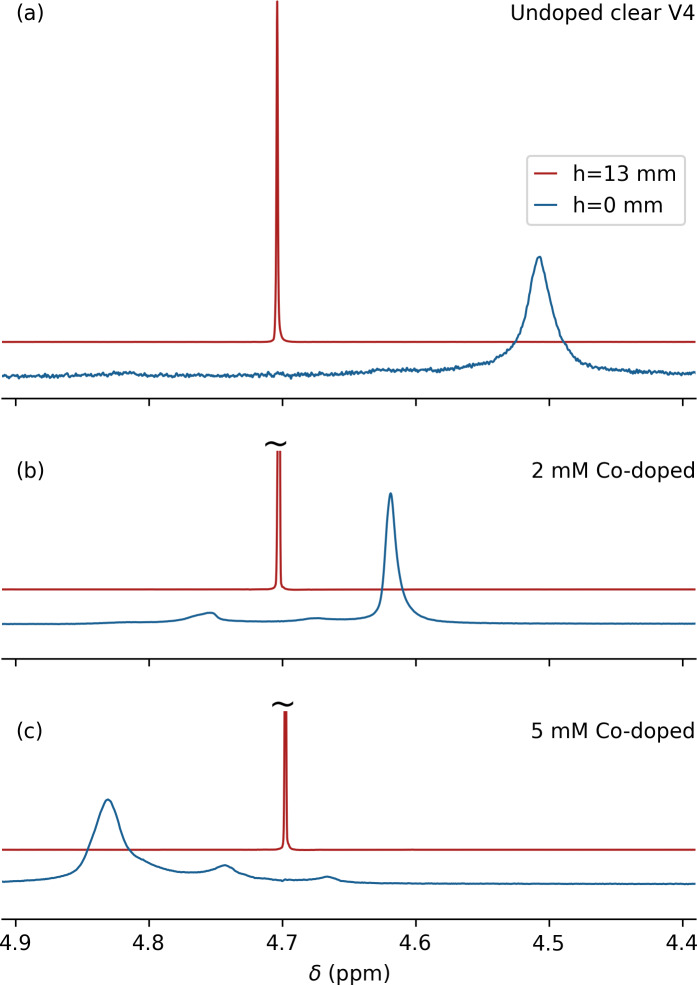
Effect of cobalt(II) 2-ethylhexanoate doping on the magnetic susceptibility of printed Formlabs Clear V4 resin. A solid plug of printed resin that is 2 cm in length and has a 4.0 mm outer diameter (OD) was inserted into a regular 5 mm OD NMR sample tube (Wilmad-507; ID 4.2 mm), prefilled with 0.7 mL 99 %D_2_O / 1 %H_2_O. Overlaid spectra are shown from 0.6 mm thickness slices centered at the top of a Clear V4 plug (blue) and 13 mm above the plug (red). **(a)** No doping, **(b)** 2 mM cobalt(II) doping, and **(c)** 5 mM cobalt(II) doping.

The frequency difference observed at the D_2_O–resin interface versus the top of the coil was -0.195 ppm (Fig. 3a), corresponding to $\chi_{\mathrm{ClearV}4}=-9.40$ ppm, which is close to the value of -9.33 ppm measured for this resin by magnetic resonance imaging (Sangal et al., 2023). Considering that a mismatch in magnetic susceptibility between the sample cell and the solution impacts the achievable $B_{0}$ homogeneity and that $\chi_{\mathrm{ClearV}4}<\chi_{H_{2}O},\chi_{D_{2}O}$ , increasing the value of $\chi_{\mathrm{ClearV}4}$ by paramagnetic doping of the resin in principle allows for the elimination of this difference. Finding a paramagnetic doping substance that is miscible with the printer resin and does not impact the performance of the 3D printer proved challenging. For example, the use of concentrated CuCl_2_ in methanol strongly impacted the polymerization kinetics. The same problem was encountered for a range of strong paramagnetic chelated substances, such as gadodiamide (Omniscan), which is commonly used in protein paramagnetic relaxation enhancement measurements and as a contrast agent in magnetic resonance imaging. However, the hydrophobic cobalt(II) complex, cobalt(II) bis(2-ethylhexanoate), available from Sigma-Aldrich as a 65 wt % solution in mineral spirits (product number 444545), proved miscible with the Clear V4 printer resin without a major adverse impact on print quality.

Comparison of the difference in HDO resonance frequencies obtained for slices at 13 mm above the interface between solvent and printed plug and at the interface for three different levels of the cobalt(II) doping, 0 mM (Fig. 3a), 2 mM (Fig. 3b) and 5 mM (Fig. 3c), shows a doping-dependent decrease from +117 to -80 Hz for the NaCl-free 99 % D_2_O sample. This result indicates that it is possible to match the susceptibility of the solvent to that of the printed resin. However, that would require a large number of printed cells to cover the ionic strength range from 0 to 2 M salt while also accounting for the difference between D_2_O and H_2_O samples.

In practice, printing sample cells with different levels of cobalt(II) doping is labor-intensive because it requires thorough cleaning of the printing vat used by the Formlabs 3D laser printer. Printing with doped resin requires mixing of the viscous resin at the molecular level with the doping agent and keeping it homogeneous during printing. The latter also required some modification of the printer to prevent refreshing the printer resin with undoped resin from a sealed cassette during printing. We therefore resorted to printing the sample cells with an ellipsoidal shape that, to a good approximation, were insensitive to the susceptibility mismatch between the solvent and printed resin (Schenck, 1996; VanderHart, 1996).

**Figure 4:**
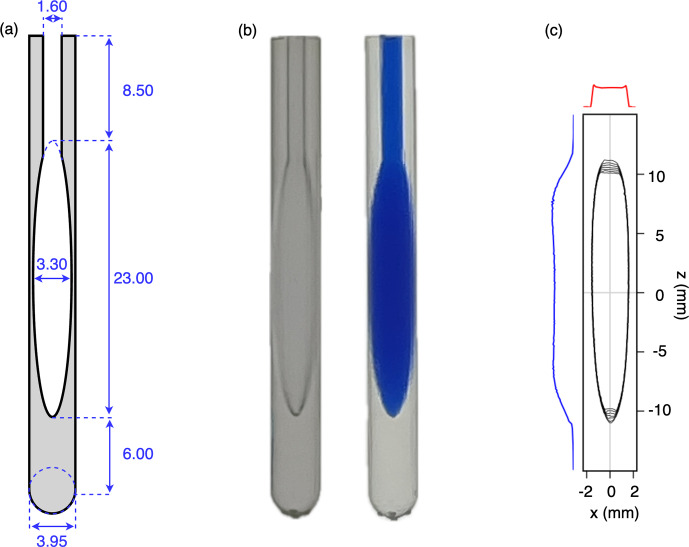
Images of the printed microcell. **(a)** Technical drawing. Note that the actual dimensions of printed material are slightly larger due to polymerization of a thin ( ∼0.15 mm) sticky surface layer that remains on the printed cell prior to subsequent UV curing. **(b)** Photographs of the printed cells (left) prior to and (right) after filling with a blue dye solution. **(c)** Contour plot of a sagittal xz cross section through a 3D image of a 97 % D_2_O, 3 % H_2_O sample containing 1.8 mM CuCl_2_ to shorten the ^1^HT_1_ value to ∼0.7 s. The image was recorded in absorption mode on an 800 MHz NMR spectrometer equipped with a three-axis pulsed field gradient probe head. The total measurement time was 9 min.

### 2.5 Performance of a Clear V4 ellipsoidal microcell

**Figure A3:**
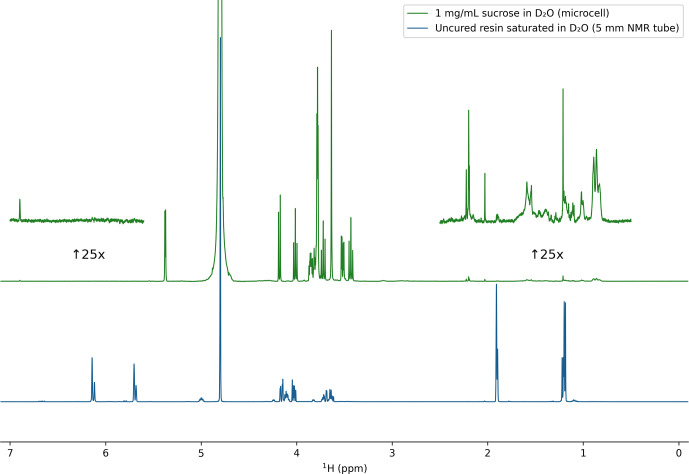
^1^H NMR spectra (500 MHz) of liquid Formlabs Clear V4 resin, saturated in D_2_O (5 mm tube, 128 scans; blue trace), and 1 mg mL^−1^ sucrose in D_2_O recorded in a 3D-printed sample cell 48 h after preparation (512 scans; green trace). The blue spectrum is scaled down 10-fold relative to the green spectrum and corresponds to ca. 10 mg mL^−1^ precursor concentration. The dissolved oligomer impurity concentration in the 3D-printed microcell sample corresponds to ca. 24 $µg\;{\mathrm{mL}}^{-1}$ . Insets correspond to the olefinic and aliphatic regions upscaled 25-fold. According to the Formlabs Clear V4 data sheet, the monomeric precursors in the blue spectrum correspond to methacrylic acid monoester with propane-1,2-diol and 7,7,9 (or 7,9,9)-trimethyl-4,13-dioxo-3,14-dioxa-5,12-diazahexadecane-1,16-diylbismethacrylate (see https://formlabs-media.formlabs.com/datasheets/1801037-SDS-ENEU-0.pdf, last access: 7 July 2025).

For applications to proteins, we settled on an ellipsoidal microcell design with a volume of 130 µL (Fig. 4a). A printed access channel 1.6 mm in diameter (measured 1.3 mm) and 8.5 mm in length and a volume of ∼11 µL was used for cleaning of the sample after the initial print and prior to further hardening of the resin in a Formlabs light chamber (see Sect. 4.2). A subsequent overnight rinse with Milli-Q H_2_O at 60 °C was used to remove small water-soluble contaminants. A comparison of the NMR spectrum of these impurities with those in a sample obtained by briefly vortexing a mixture of unpolymerized resin and water followed by entering the aqueous phase into an NMR sample tube (Fig. A3) suggests that these impurities consist of residual, methacrylate-based small oligomeric species that apparently can diffuse from the cell walls into the aqueous contents of the cell. Leaving the sample cells filled with H_2_O for a week prior to a final rinse reduces the impurity levels further but, in our experience, is not necessary considering we have not observed any interaction between these very low impurity concentrations and isotopically enriched proteins. For applications to samples in D_2_O, leaving the microcell filled with D_2_O for 24 h, prior to using it, reduces the intensity of a weak, very broad ( ∼1 kHz) signal at ∼3.8 ppm that results from H_2_O diffusing into the resin.

Because the digital printer increases the size of printed parts by a small amount due to partial polymerization adjacent to the laser-selected spots, the printed walls of the chamber are actually slightly thicker than designed, and the total volume of the cell, including its access channel, was measured gravimetrically to be 130 µL (Fig. 4b).

A sagittal ( xz ) cross section through the absorption-mode 3D image of the cell recorded on a Bruker Neo 800 MHz instrument equipped with a three-axis pulsed field gradient probe head yielded a shape that matched the ellipsoidal design (Fig. 4c), but that did not include the access channel because the solvent in that channel falls outside the RF coil. Small distortions near the bottom of the image correspond to the dropoff in RF coil receptivity. Distortions at the top of the sample originate from the access channel which also distorts the ellipse. However, cross sections taken through the 3D image along the x and z axis through the center of the sample plotted along the sides of the 3D image show the expected nearly rectangular shape, indicative of linear imaging gradients.

**Figure 5:**
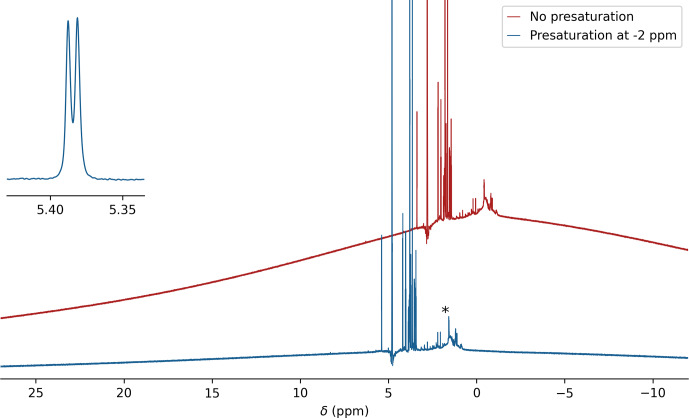
^1^H background of the printed microcell. The regular ^1^H NMR spectrum of a 1 mg mL^−1^ solution of sucrose in 99 % D_2_O recorded without presaturation (red) and with ^1^H presaturation at -2 ppm (blue) using a 100 Hz RF field strength to suppress the ^1^H background of the solid resin. Resonances from small impurities released from the printed cell when it has only briefly ( ∼2 h) been rinsed with H_2_O are marked with an asterisk. However, a subset of these resonances very slowly reappear over a period of multiple days at concentrations much lower than seen in the figure (see Fig. A3). For display purposes, the not-presaturated spectrum has been shifted upfield by 2 ppm and offset vertically. The inset shows the splitting for the sucrose anomeric doublet at 5.39 ppm.

^1^H non-spinning line widths of ∼1 Hz at half height (600 MHz) obtained with the cell (expanded anomeric doublet in Fig. 5) were slightly larger than those obtained for a regular 5 mm NMR sample in the same probe head. With a width of only ∼13 Hz at 0.55 % of the HDO peak height, the line shape is also very good, which is the most important for protein NMR studies, where protein ^1^H line widths at half height commonly exceed 10–20 Hz due to fast transverse relaxation and the absence of a “hump” in the water line shape is critical for good solvent suppression.

We note that sometimes microscopic air bubbles can form inside the cell after filling it with the NMR sample solution. When this happens, it has a very strong adverse impact on both line shape and line width. To eliminate the potential presence of such air bubbles, we briefly ( ∼20 s) spin the filled sample cell in a SpeedVac, operating at a pressure of ca. 150 Pa, and replenish any lost volume by adding a few microliters of the protein solution to the access channel.

For observation of simple 1D ^1^H spectra, without echo delays, the protons of the resin yield a strong background signal that is broad due to the rapid transverse relaxation of this solid material. This background signal is readily suppressed by spin echo delays prior to the start of signal acquisition, as are already present in nearly all protein NMR experiments. It can also be effectively reduced by presaturating this broad background with a weak RF field outside the spectral region of interest. Saturation with a 100 Hz RF field, applied at -2 ppm in the ^1^H spectrum, attenuates the background signal by about 6-fold (Fig. 5).

**Figure 6:**
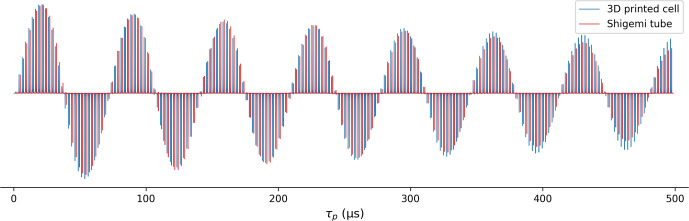
Comparison of ^1^H RF field homogeneity in a 280 µL Shigemi sample cell (red) and in the 3D-printed 130 µL 23×3.3 mm ellipsoidal microcell (blue). Signal intensity is shown as a function of ^1^H pulse duration ranging from 0.2 to 497.7 µs , generated by the Bruker macro paropt. Both samples contained the same solution of the PBS buffer in 99 % D_2_O. The RF power for the microcell sample was adjusted to be 2.7 dB lower than for the Shigemi sample to equalize the 90° pulse lengths.

The ^1^H RF field homogeneity was compared for the microcell and for a Shigemi sample (straight wall) containing 280 µL of phosphate-buffered saline (PBS) solution in 99 % D_2_O. Due to the substantial detuning of the 600 MHz cryoprobe used for this work by the ionic solution in the Shigemi sample, the RF power for this sample was increased by 2.7 dB over the power used for the microcell to yield the same 90° pulse width of ∼17 µs . The comparison of the decay of the signal when the excitation pulse is increased from 0.2 to 497.7 µs using the Bruker paropt module (Fig. 6) shows slightly better RF field homogeneity for the smaller 3D-printed microcell than for the Shigemi sample. Notably, the intensity of the signal after a 90° pulse was only ∼10 % lower for the printed microcell than for the Shigemi tube that contained more than double the volume of the same solution.

### 2.6 Observation of high-resolution protein NMR spectra

The high resolution and sensitivity obtained with the microcell are illustrated for two proteins, N-acetylated α -synuclein and native C-terminal amidated melittin in its monomeric and tetrameric forms. Complete or nearly complete N acetylation of α -synuclein is invariably present in mammalian cells (Bartels et al., 2011) and strongly impacts its interaction with phospholipids (Kang et al., 2012; Maltsev et al., 2012). By simultaneously including a plasmid for expressing the NatB complex, needed for acetylation of α -synuclein, together with a plasmid for α -synuclein, fully N-acetylated protein can also be obtained from bacterial expression systems (Johnson et al., 2010). Although this combined expression reduced protein yields in our hands, it enabled the pivotal incorporation of stable isotopes, such as ^15^N, in the biologically relevant state of the protein.

**Figure 7:**
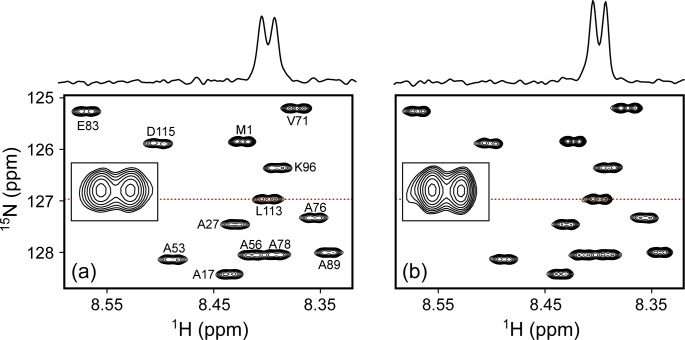
Comparison of small regions of the 600 MHz ^1^H-^15^N HSQC spectra of 70 µM N-acetylated α -synuclein at 20 °C in the PBS buffer, pH 6.5. Each spectrum results from $175^{*}\times 4000^{*}$ data points with two transients per free induction decay (FID) for total measuring times of 21 min each. **(a)** 130 µL in the 3D-printed microcell. **(b)** 500 µL in a standard 5 mm NMR tube. Lowest contours are drawn at the same level above the respective RMS noise. Insets show the expansion of the L113 cross peak, with cross sections through L113 above the panels at locations marked by the red dotted lines. The comparison shows slightly lower resolution of the ^1^H^*N*^-^1^H^*α*^ doublets for the microcell, reflected in 12 % lower S/N versus 6.5 % lower peak integrals relative to RMS noise.

As can be seen, in ca. 20 min, a high-quality gradient-enhanced ^1^H-^15^N HSQC spectrum was obtained for 130 µg of N-acetylated α -synuclein (14.5 kDa) in PBS buffer, pH 6.5 when using the microcell, which approaches the sensitivity and resolution that was obtained for 500 µg protein in a regular 5 mm sample cell using a 0.5 mL sample volume (Fig. 7).

**Figure 8:**
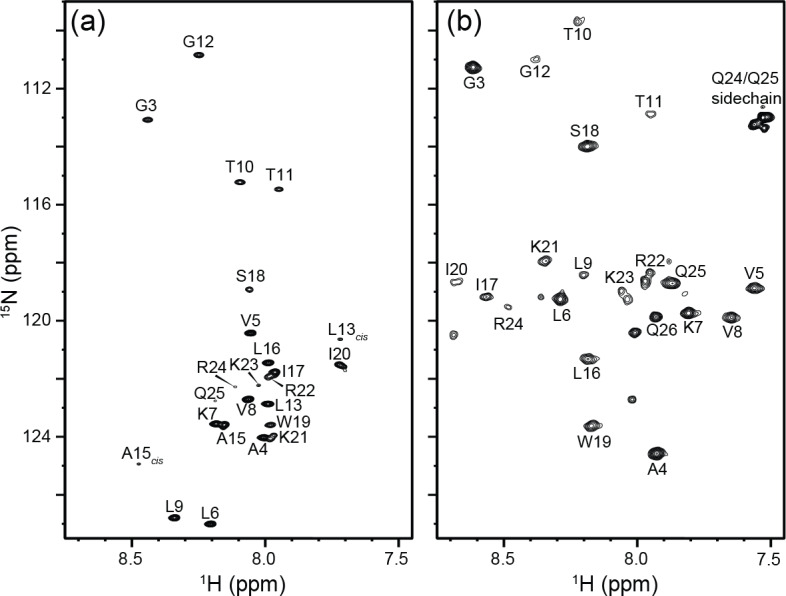
800 MHz ^1^H-^15^N HSQC spectra from 90 µg (250 µM ) of ^15^N-labeled native melittin in a 130 µL microcell. Both spectra were collected at 288 K in 10 mM sodium phosphate buffer, pH 7.0, containing 3 % D_2_O. **(a)** Sample containing no NaCl, where melittin remains an intrinsically disordered monomer. L13_cis_ and A15_cis_ correspond to residues in monomers with P14 in the *cis* conformation. The data were collected with two transients per FID using 200* points in the indirect dimension corresponding to an evolution of 103 ms and a total measurement time of 27 min. **(b)** 15.2 mg of NaCl was added to the sample from **(a)** to reach a final concentration of 2 M NaCl. Under these conditions, melittin adopts an α -helical tetrameric conformation. The data were collected with eight transients using 100* points in the indirect dimension ( $t_{1,\max}=51$ ms) and a total experimental time of 52 min.

The utility of the microcell becomes even more compelling for the study of the tetramerization of native melittin, which requires C-terminal amidation. The latter involves a large number of chemical derivatization and purification steps (Gelenter and Bax, 2023), making it very challenging to generate adequate quantities of peptide for driving it to its tetrameric state within a standard 5 mm NMR tube. Tetramerization is promoted by increasing the NaCl concentration, but the tuning of the RF circuitry in cryoprobes often limits salt concentrations to be below ca. 0.5 M. Here, we demonstrate that the microcell enables observation of the monomer-tetramer equilibrium at salt concentrations of 2 M and that indeed even at a moderate peptide concentration of 250 µM , the peptide is fully tetrameric in the presence of 2 M NaCl (Fig. 8). Interestingly, residues L9–R24, close to the center of the peptide, are attenuated by an exchange process that is much less prevalent at low ionic strength and high peptide concentration (Gelenter et al., 2024). We speculate that the line-broadening associated with these weaker resonances arises from the exchange process between the asymmetric arrangement of the two dimers seen in its X-ray tetrameric structure (Terwilliger and Eisenberg, 1982) switching from fast exchange at lower ionic strength to intermediate exchange at 2 M NaCl.

Notably, the same microcell was used for the two melittin spectra. After recording of the low-ionic strength spectrum, the content of the microcell was removed and used to dissolve 15.2 mg NaCl prior to the insertion of the high-ionic-strength sample into the original cell, thereby demonstrating the recyclability of the tube.

## 3 Concluding remarks

3D printing enables the efficient and relatively inexpensive creation of complex, customized products with minimal waste. It is extensively used for prototyping new designs of items with intricate or complex geometries and has a broad impact on science and engineering. 3D printing also enabled the design and construction of high-performance solid-state NMR probes, offering similar or improved filling factors due to the coil being in close proximity to the sample, resulting in high RF transmit-and-receive efficiencies (Long et al., 2021; Pereira et al., 2023).

In solution NMR spectroscopy, 3D-printed bioreactor platforms were introduced that are compatible with low-field NMR spectrometers that accommodate bioengineered 3D cell models (Mangas-Florencio et al., 2025). That work consisted of a bioreactor made of biocompatible materials and included a microfluidic system for optimization of cell culture conditions during the actual NMR data collection process.

The application of 3D printing to high-field solution NMR spectroscopy has remained rather limited, largely due to the requirements of high magnetic field homogeneity and minimal background signals. Our study demonstrates that the homogeneity requirement can be met by printing small sample cells with an ellipsoidal shape. The half-height line width achievable for our microcell is ca. 1 Hz and remains limited by the precision at which the cell's surface can be printed. For shimming purposes, we first used Bruker's topshim program to adjust field homogeneity to starting values on a regular-solution NMR sample filled to the same height ( ∼40 mm) as the length of the microcell. Subsequently, after entering the microcell into the magnet, we used topshim followed by iterative tuning of z , $z^{2}$ , x , y , xz , and yz gradients. The microcells can easily be recycled as they slide in and out of standard high-quality Wilmad-507, New Era NE-HP5, or Norell Standard Series 5 mm NMR tubes. The access channel of the microcell is sufficiently small that surface tension prevents the aqueous solution from leaving the microcell when the 5 mm NMR tube is fully inverted, while the microcell slides out of it.

Although inexpensive, sample cells are easily recycled as highlighted for the melittin sample, where 15.2 mg of salt was added to the initial sample by first removing the sample solution, dissolving the NaCl, and re-injecting the solution into the original cell, all with minimal losses. It is advisable to briefly spin and expose the sample to vacuum to remove dissolved gasses, in particular for lengthy experiments. Formation of even microscopic air bubbles deteriorates homogeneity for the microcell sample more than for the Shigemi or larger conventional NMR sample tubes.

The sample cell appears unsuitable for the use of organic solvents which dissolve and release resin components, resulting in strong narrow background signals. Even when using water as the solvent, slowly increasing signals from micromolar quantities of resin-derived small molecules appear in the ^1^H spectrum over a period of days (marked by an asterisk in Fig. 5). However, the standard use of isotope-enriched multi-dimensional multinuclear NMR experiments keeps these resonances well below the signal-to-noise threshold level, and unless such contaminants have a strong interaction with the protein studied they have no effect on the acquired spectra. We note that commercial 3D-printing resins are formulated in a way that tries to meet various performance specifications, such as material strength, printing resolution, and printing speed. As such, they may not be optimal for high-precision work, such as that required for protein NMR. Future development of resins is needed to allow for high-precision printing while minimizing release of small, incompletely polymerized precursors into the aqueous phase.

While a small amount of solvent ( <0.1 %) can diffuse into cavities of the polymerized resin, no detectable loss of solute signal was observed. For example, the intensity of the sucrose NMR resonances remained unchanged to within ±0.2 % over a duration of more than 1 week.

We cleaned the microcells by soaking them overnight at 60 °C in Milli-Q water and subsequently removing most of the solvent with a standard gel micropipette tip that did not quite reach the bottom of the cell, which is followed by upside-down centrifugation after insertion into an Eppendorf tube to remove the remainder of the solvent. An additional rinse with 130 µL D_2_O followed by centrifugation and vacuum exposure can be used to remove any residual solvent protons if the sample is intended for measurements in highly deuterated D_2_O. These are the most labor-intensive steps in preparing the sample cells, but limited quantities of unrinsed sample cells are available upon request.

## 4 Methods

### 4.1 Magnetic susceptibility measurements

Selective excitation while applying a 11.5 G cm^−1^ (20 % on the Bruker Neo-600 instrument) was used for collecting the HDO solvent resonance measurements (Sect. A1) of 0.6 mm thickness slices at various heights, h , above the flat interface between a solid printed plug and the aqueous solvent. The printed plug with an outer diameter of 4 mm and a length of 20 mm, including its hemispheric bottom, was inserted and pushed to the bottom of a regular NMR tube prefilled with 0.7 mL of 97 % D_2_O or 97 % H_2_O solution. A similar tube without the plug was filled to the same height with the same solution and used for shimming the magnetic field using topshim prior to inserting the sample with the plug at its bottom, where the same shim settings of the sample without the plug were used. The thickness of the aqueous fraction of the slice collected for h=0 was 2-fold smaller than for slices at h>0.3 mm, with a correspondingly lower-integrated volume. The frequency of slices collected at heights >∼10 mm above the plug became essentially independent of h . The slice collected at h=0 shows extensive line broadening due to the large field gradient at the solvent–plug interface (Fig. 2a–c). The difference in parts-per-million frequency was used as a measure for $(\chi_{\mathrm{solvent}}-\chi_{\mathrm{resin}})/2$ .

### 4.2 Printing of the microcell

Cells were printed in Clear V4 resin on a Form 3+ printer at 25 µm resolution to achieve a smooth surface finish. The designs were created in OpenSCAD (2021.01) using standard STL export settings and then prepared for printing in PreForm (3.43.2). Models were oriented so that the bottom of each cell faced downward on the build platform, with 0.2 mm touch point supports attached only at the bottom of the cells. The cells were washed with 15 mL isopropyl alcohol (IPA) at a rate of 5 mL min^−1^ through a syringe needle (0.58 mm ID) with a blunt tip, connected to a fast protein liquid chromatography (FPLC) pump. Cells were kept vertical during printing and subsequent UV curing using Form Cure for 16 h at 60 °C. Subsequently, cells were filled with Milli-Q water and immersed in a water-filled falcon tube that was heated at 60 °C for 12 h to remove water-soluble components. After this cleaning, to further minimize the amount of resin-derived impurities from leaching from the cell wall into the solvent, which occurs at a rate that decreases steadily with time, cells can be stored filled with and immersed in water for a week at room temperature. After the removal of the water from the cells by pipetting followed by centrifugation upside down in Eppendorf tubes, they were briefly dried in a vacuum. Subsequent use of such a cell showed strongly reduced intensities of these impurity signals (Fig. A3).

The paramagnetically doped resin was prepared by mixing Formlabs Clear V4 resin with a cobalt(II) bis(2-ethylhexanoate) solution (65 wt % in mineral spirits, Sigma-Aldrich, 444545) on a shaker at 37 °C and 200 rpm for 30 min. The plugs printed from doped resin were washed with IPA and cured at 60 °C for 16 h using Form Cure.

### 4.3 NMR sample preparation

The microcells were filled to the top with the sample solution ( ∼130 µL ) using a gel-tip pipette and degassed at ca. 150 Pa pressure for 20–30 s using a SpeedVac (Savant, SVC-100-H). After degassing, the cells were topped off with ca. 2 µL of additional sample solution and inserted into standard Wilmad-507, New Era NE-HP5, or Norell Standard Series 5 mm NMR tubes.

### 4.4 Imaging of the microcell

Although MRI normally uses absolute value mode displays, higher-resolution absorption mode spectra can also be obtained (Bretthorst, 2008). For generating images of the microcell, we used a very simple one-pulse sequence with variable durations of the x , y , and z gradients for encoding the three spatial dimensions (Sect. A4). The experiment used Rance–Kay quadrature selection (Palmer et al., 1991; Kay et al., 1992) in both the x and y dimensions by collecting four scans per hypercomplex time domain data point (ns = 1). NMRPipe (Delaglio et al., 1995) processing of the 3D time domain matrix was used to generate regular, amplitude-modulated quadrature (States et al., 1982) in both indirect dimensions (see Sect. A5 for NMRPipe processing script). A total of 20⋅(x)×20⋅(y)×512⋅(z) data points were collected with a total acquisition time of ca. 9 min for a sample that contained 1.8 mM CuCl_2_ in 99 % D_2_O.

## Figures and Tables

**Figure A1:**
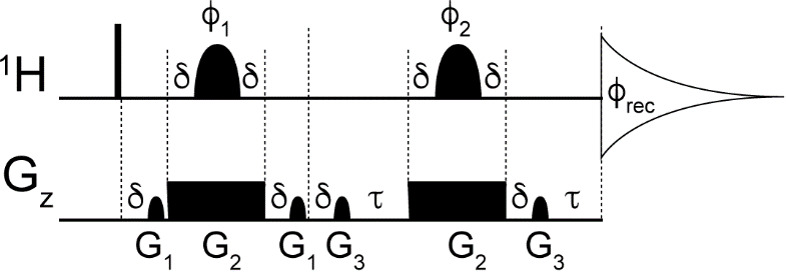
Diagram of the slice-selection pulse sequence used to measure magnetic susceptibility. The narrow bar represents the 90 ^1^H excitation pulse. The shaped pulses $\varphi_{1}$ and $\varphi_{2}$ have a profile of Reburp (Geen and Freeman, 1991) with a duration of 2 ms and a variable offset frequency ranging from ca. +23 to -30 kHz from the water signal resonating at 4.7 ppm. The gradient pulses are either sine-bell-shaped or rectangular, with durations of 0.5, 2.1, and 0.5 ms and a strength of 2, -11.5 , and 3.5 G cm^−1^ for G_1_, G_2_, and G_3_, respectively. Delays: δ=50 µs and τ=60 µs . The following phase cycling scheme was used: $\varphi_{1}=y$ , y , y , y , -x , -x , -x , -x , -y , -y , -y , -y , x , x , x , x ; $\varphi_{2}=x$ , y , -x , -y ; $\varphi_{\mathrm{rec}}=x$ , -x , x , -x , -x , x , -x , x . The interscan delay was set to 4 s, number of scans to 4, spectral width to 8620 Hz, and acquisition time to 2 s.

**Figure A2:**
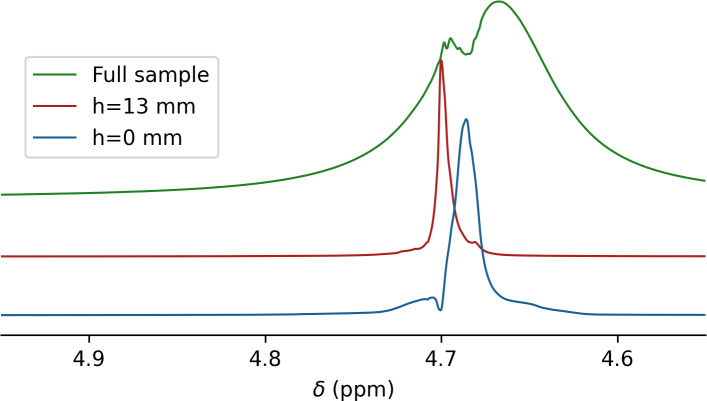
Same as Fig. 1b (main text) but for a solution containing 97 % H_2_O / 3 % D_2_O. The resonance of the full Shigemi tube sample (green) is broadened by radiation damping and shown together with slices taken at h=0 mm (blue) and at h=13 mm (red). The center of the green resonance is shifted relative to its regular position due to the radiation damping effect in the deliberately mistuned probe (Torchia, 2009).

## Data Availability

Scripts used to generate figures and the associated processed spectral data are available at 10.5281/zenodo.15858288 (Kakeshpour et al., 2025b).

## Appendix A Supplementary data and code

### A4 Bruker pulse program used for imaging the microcell

#include<Avance.incl>
#include<Grad.incl>
#include<De.incl>

"d11=30m"

"in0=inf1". ;125u
"in10=inf2" ;125u

"d0=0"
"d10=0"

define list<gradient> EA2 = { 1.000 -1.000}

aqseq 321

1 ze
  1m

2 d11 groff
  10u BLKGRAD
  10u pl9:f1 ;set to 1000dB unless presat is needed
  10u fq=cnst2(bf ppm):f1 ;chemical shift for the resin to be presat'ed
  d1 cw:f1
  10u do:f1
  10u UNBLKGRAD
  p20:gp20. ;3m at 33% of z-grad
  1m fq=0:f1 ;shift carrier back to be on-resonance with water
  1m pl1:f1
  (p1 ph1):f1 ;20 degree flip angle
  1u gron1*EA ;x-grad 8%
  d0
  1u groff

  1u gron2*EA2 ;y-grad 8%
  d10
  1u groff
  600u ;delay to dephase a very broad hump
  1u gron3 ;z-grad -1%
  go=2 ph31
  d11 groff mc #0 to 2
  F1EA(calgrad(EA), caldel(d0)) ;TD1=40
  F2EA(calgrad(EA2), caldel(d10)) ;TD2=40
  d11 BLKGRAD
exit

ph1=0
ph31=0

### A5 NMRPipe conversion and processing scripts

#!/bin/csh

bruk2pipe -verb -in ./ser \
-bad 0.0 -ext -aswap -DMX -decim 1240 -dspfvs 20 -grpdly 68 -ws 8 -noi2f \
  -xN 1024 -yN 40 -zN 40 \
  -xT 512 -yT 20 -zT 20 \
  -xMODE DQD -yMODE Echo-AntiEcho -zMODE Echo-AntiEcho \
  -xSW 16129.032 -ySW 8000.000 -zSW 8000.000 \
  -xOBS 800.134 -yOBS 800.134 -zOBS 800.134 \
  -xCAR 4.821 -yCAR 4.821 -zCAR 4.821 \
  -xLAB Hz -yLAB Hy -zLAB Hx \
  -ndim 3 -aq2D Complex \
| pipe2xyz -x -out ./fid/test%03d.fid -ov

xyz2pipe -in fid/test%03d.fid -x \
| nmrPipe -fn SP -off 0.5 -end 0.98 -pow 2 -c 0.5 \
| nmrPipe -fn ZF -zf 2 -auto \
| nmrPipe -fn FT \
| nmrPipe -fn PS -p0 149 -p1 38 -di \
| nmrPipe -fn TP \
| nmrPipe -fn SP -off 0.5 -end 0.98 -pow 1 -c 0.5 \
| nmrPipe -fn ZF -zf 2 -auto \
| nmrPipe -fn FT \
| nmrPipe -fn PS -p0 135 -p1 0 -di \
| nmrPipe -fn ZTP \
| nmrPipe -fn SP -off 0.5 -end 0.98 -pow 1 -c 0.5 \
| nmrPipe -fn ZF -zf 2 -auto \
| nmrPipe -fn FT \
| nmrPipe -fn PS -p0 90 -p1 0 -di \
| nmrPipe -fn TP \
| nmrPipe -fn POLY -ord 4 -nw 300 -nl 301 1740 \
| pipe2xyz -out ft/test%04d.ft3 -x -ov -verb

## Code availability

The STL file with the code for printing the cells is available at 10.5281/zenodo.15064675 (Kakeshpour et al., 2025a).
